# Hydrogen Oxidation Pathway Over Ni–Ceria Electrode: Combined Study of DFT and Experiment

**DOI:** 10.3389/fchem.2020.591322

**Published:** 2021-02-01

**Authors:** Yunan Jiang, Shuang Wang, Jun Xu, Minghao Zheng, Yi Yang, Xiaojun Wu, Changrong Xia

**Affiliations:** ^1^Chinese Academy of Sciences (CAS) Key Laboratory of Materials for Energy Conversion, Department of Materials Science and Engineering, University of Science and Technology of China, Hefei, China; ^2^Department of Materials Science and Engineering, University of Science and Technology of China, Hefei, China; ^3^Hefei National Laboratory for Physical Science at the Microscale, University of Science and Technology of China, Hefei, China

**Keywords:** Ni-ceria cermet, anode reaction kinetics, solid oxide fuel cell, density functional theory, hydrogen spillover

## Abstract

Ni–ceria cermets are potential anodes for intermediate-temperature solid oxide fuel cells, thanks to the catalytic activity and mixed conductivities of ceria-based materials associated with the variable valence states of cerium. However, the anodic reaction mechanism in the Ni–ceria systems needs to be further revealed. Via density functional theory with strong correlated correction method, this work gains insight into reaction pathways of hydrogen oxidation on a model system of Ni_10_-CeO_2_(111). The calculation shows that electrons tend to be transferred from Ni_10_ cluster to cerium surface, creating surface oxygen vacancies. Six pathways are proposed considering different adsorption sites, and the interface pathway proceeding with hydrogen spillover is found to be the prevailing process, which includes a high adsorption energy of −1.859 eV and an energy barrier of 0.885 eV. The density functional theory (DFT) calculation results are verified through experimental measurements including electrical conductivity relaxation and temperature programmed desorption. The contribution of interface reaction to the total hydrogen oxidation reaction reaches up to 98%, and the formation of Ni–ceria interface by infiltrating Ni to porous ceria improves the electrochemical activity by 72% at 800°C.

## Introduction

As a device that efficiently transfers chemical fuel energy into electricity, solid oxide fuel cell (SOFC) is given a competitive edge in long-term stability, fuel flexibility, and sustainable energy in the future (Ormerod, 2003). One of the key steps in the electrochemical energy conversion process is the fuel oxidation reaction at the anode, where the fuel molecule such as H_2_ and CO combines with an oxygen ion $O_{O}^{\times }$ meanwhile releasing electrons and forming an oxygen ion vacancy $V_{O}^{••}$. In the case where hydrogen or reform gas is supplied as the fuel, the hydrogen oxidation reaction at the anode can be written as Equation (1).

(1) $$\begin{array}{r} H_{2}\left(g\right)+O_{O}^{\times }\to H_{2}O\left(g\right)+V_{O}^{\cdot \cdot }+2e\prime \end{array}$$

The equation shows that the anodic reaction usually takes place at the three-phase boundaries (3PB), where the fuel gas, the electrons, and oxygen ions are all available (Sun and Stimming, 2007). This is the case for the state-of-the-art Ni–yttrium-stabilized zirconia (YSZ) cermet anodes, which have been widely used in intermediate- and high-temperature SOFCs that operate at temperature up to 1,000°C (Horita et al., 2006). In the Ni–YSZ cermet, the reaction occurs only at the Ni–YSZ–gas 3PB since YSZ is a pure oxygen ion conductor that transport oxygen ions and oxygen vacancies while metallic nickel plays the role of both electronic conductor and electrocatalyst.

Different from YSZ, ceria-based oxygen ion conductors also have electronic conductivities due to the facile conversion between Ce^3+^ and Ce^4+^ oxidation states in the anodic conditions of low oxygen partial pressure and high temperature. The variable oxidation state dedicates ceria to catalyze the fuel oxidation reaction and facilitates the formation of additional oxygen vacancies. The character of mixed conductivity makes it possible to electrochemically oxidize the fuel on the ceria surfaces. Thus, replacing zirconia with ceria will extend the reaction sites from 3PB to the whole surface of the cermet anode within the effective reaction zone and consequently increase the electrode performance. In addition, the oxygen ionic conductivities of ceria-based electrolytes such as samaria-doped ceria (SDC) are several times higher than those of zirconia-based electrolytes like YSZ. The high conductivity increases the availability of oxygen ions, thus enlarging the electrochemically effective zone. Comprehensively, compared with Ni–YSZ, ceria-based cermets exhibit much higher anodic activity, especially at intermediate temperatures below 800°C (Kašpar et al., 1999; Fergus, 2006). Besides, when hydrocarbon fuel is supplied to the anode, Ni–ceria cermets show higher resistance to carbon deposition than Ni–YSZ since carbon can be removed through combining with oxygen from ceria due to the capability of changing oxidation states (Ramirez-Cabrera et al., 2000; Laosiripojana et al., 2005).

While Ni–ceria shows many advantages, its anode reaction mechanism is not understood as clearly as that of Ni–YSZ. Hydrogen oxidation occurs only at the 3PB in the system of Ni–YSZ, and the kinetic pathway for such reaction could be subdivided into three parts: hydrogen adsorption on Ni, hydrogen spillover to the Ni–YSZ interface, and water formation and desorption at 3PB (Shishkin and Ziegler, 2009; Cucinotta et al., 2011). For the Ni–ceria cermets, the reaction takes place not only at 3PB but also at the ceria surface due to the higher availability of surface oxygen vacancy. By comparing the electronic properties of Ni–YSZ and Ni–CeO_2_ interfaces, Shishkin and Ziegler demonstrate that the formation of the surface vacancy is much easier in the case of Ni–CeO_2_, while oxygen vacancy can only exists at 3PB for Ni–YSZ (Shishkin and Ziegler, 2014). Calculation on Ni–CeO_2_(111) system reveals that the surface oxygen vacancy is energetically more favored than the interfacial oxygen vacancy (Shishkin and Ziegler, 2010a). Furthermore, Hahn et al. suggest that oxygen vacancy is affected by the position of reduced Ce^3+^ sites in CeO_2_ and the quantum number of their occupied *f*-type orbitals (Hahn et al., 2015). Although some properties in Ni–CeO_2_ system are studied through theoretical calculations, the overall reaction pathway of hydrogen oxidation is still uncovered. In this work, we adopt density functional theory with strong correlated correction (DFT + U) method to evaluate different molecular pathways of H_2_ oxidation on Ni–CeO_2_(111) surface. Possible H_2_ oxidation mechanisms with different pathways is unveiled and demonstrated systematically. The energetically favored pathway is concluded from theoretical calculations and further shown *via* experimental approaches.

## Computational Methods

All spin-polarized calculations were accomplished by DFT method implemented in the Vienna *ab initio* simulation package (VASP) (Kresse and Furthmüller, 1996). The generalized gradient approximation (GGA) with the Perdew–Burke–Ernzerhof (PBE) functional was used (Blochl et al., 1994; Perdew et al., 1996; Hammer et al., 1999). To accurately describe the strongly corrected electrons of the localized Ce 4f orbitals, GGA + U with U_eff_ = 5 eV for Ce ions was adopted (Nolan et al., 2005; Andersson et al., 2007). The projector-augmented wave (PAW) method was carried out to deal with the interaction between ionic core electrons and valence electrons with Ce([Xe]4f^1^5d^1^6s^2^), Ni([Ar]3d^8^4s^2^), O(1s^2^2s^2^2p^4^), and H(1s^1^) (Blochl et al., 1994; Perdew et al., 1996). The force on atom was smaller than 0.03 eV/Å for the geometric optimization. It was set at 400 eV for the kinetic energy cutoff and the convergence criteria was 10^−4^ eV for the electronic structure calculations. The low-index (111) surface of CeO_2_ is the most stable surface, which was selected as the substrate (Conesa, 1995).

On the basis of the Monkhorst–Pack scheme, the Brillouin zone was sampled with a 6 × 6 × 6 *k*-point grid for CeO_2_ bulk. The optimized lattice parameter of ceria was 5.437 Å, consistent with the experimental result of 5.411 Å (Kümmerle and Heger, 1999). The CeO_2_(111) with a 4 × 4 slab was simulated by using two O–Ce–O triple layers of which atoms in the bottom O–Ce–O layer were fixed as their bulk positions. The thickness of vacuum was set to 15 Å to avoid the interslab interaction between two neighboring slabs along the direction perpendicular to the surface. The Ni_10_ cluster and the bond lengths of the H_2_ and O_2_ molecules have been previously optimized within a 15-Å cubic box. The evaluated that the bond lengths of the H_2_ and O_2_ molecule agree with our previous research (Wang et al., 2016). The energy of triple oxygen was utilized for all the calculations. The detailed location of H_2_ oxidation reaction and energy of the transition state (TS) were simulated with the climbing image nudged elastic band (CI-NEB) method. In the reaction pathway diagrams (Figures 3–6), Δ*E*_*x*_ represents the energy difference of structures between two neighboring reaction stages, which is equal to the energy of the *x*th stage minus the energy of the previous stage. Δ*E*_TSx_ represents the energy barrier of the transition state.

## Experimental

### Electrical Conductivity Relaxation Measurement

The reaction kinetics was measured using the electrical conductivity relaxation (ECR) method (Wang et al., 2012). CeO_2_ powder was prepared using the carbonate coprecipitation method with cerium nitrate hexahydrate [Ce(NO_3_)_3_·6H_2_O, ≥99.9%] as the precursors (Ding et al., 2008). The powder was uniaxially dry pressed under 320 MPa into rectangular bars and sintered at 1,550°C for 5 h in air. The bar density was 96.8%, measured using the Archimedes method. The bar size was about 30.0 × 5.20 × 0.52 mm^3^. Ni film was deposited on the bar surface using nickel target (4 N purity, Kejing Materials Technology) by a sputter coater (JFC-1600, JEOL) at 20 mA and under a vacuum of 8 Pa. Afterwards, the bar was heated at a rate of 3°C min^−1^ to 800°C for 2 h in reducing atmosphere to form Ni particles on the bar surface. The deposition was conducted for 20, 40, and 80 s to vary the film thickness and, consequently, the amount of Ni particles per unit area. The surface microstructures were revealed using scanning electron microscopy (SEM, JSM-6700F, JEOL). The area covered by the Ni particles and the length of Ni–CeO_2_ boundary were statistically determined using software Image J. The ECR experiments were conducted at 800°C with a digital multimeter (2001-785D, Keithley) using the four-probe technique (Wang et al., 2012). The atmosphere was switched from H_2_/Ar (5:95) to H_2_/Ar (10:90) to simulate the H_2_ oxidation reaction. The gas was humidified within a moisture bottle at room temperature, containing about 3% H_2_O, and the gas flowing rate was 200 ml min^−1^.

### Temperature Programmed Desorption/Reduction Tests

The reaction kinetics was further investigated using H_2_-temperature programmed desorption (TPD), which was performed using Thermo Electron Corporation TPDRO1100 flow apparatus. The H_2_ consumption was measured by a TCD detector. Before the TPD test, a pretreatment process for CeO_2_ and Ni–CeO_2_ bars was carried out using H_2_/N_2_ (5:95) gas mixture from room temperature to 150°C. Then, TPD signal was recorded while the samples were blown with N_2_ at a flux of 20 ml min^−1^ and heated from room temperature to 1,000°C with a heating rate of 10°C min^−1^.

### Electrochemical Measurement

The electrochemical performance was investigated using symmetrical cells composed of YSZ (8 mol% yttria-stabilized zirconia) as the electrolyte substrates and porous CeO_2_ impregnated with 8 wt% NiO as the electrodes. Dense YSZ substrates were fabricated by uniaxially pressing the 8 wt% YSZ powder (TZ-8Y, Tosoh Co., Japan) under 320 MPa followed by sintering at 1,500°C for 5 h in air. Porous CeO_2_ was prepared using printing and sintering processes. The CeO_2_ slurry was prepared by mixing the CeO_2_ powder with α-terpineol as solvent and ethyl cellulose as the binder. The slurry was printed on both sides of the YSZ substrates and then heated with the substrates at 1,100°C for 2 h to form symmetric structures with the YSZ electrolyte sandwiched in two porous CeO_2_ layers. Nickel was deposited by impregnation process. Ni(NO_3_)_2_ (≥99.9%) was dissolved in a mixture of water and ethanol (1:1 volume ratio) with a concentration of 0.5 mol L^−1^. The solution was dropped on the porous electrodes, dried and heated at 800°C in air for 1 h to form NiO particles within the porous CeO_2_ backbones. NiO was *in situ* reduced to Ni and thus forming the Ni–CeO_2_ cermet electrodes. The volume ratio of Ni to CeO_2_ was 5.5:100. The Solartron Frequency Response Analyzer 1260 and a Solartron Electrochemical Interface 1287 were used for electrochemical measurements. Ag paste (SRISR DAD-87) was used as electron collector layer, and Ag wires (≥99.9%, Xiyu Electrical and Mechanical Technology) were used to ensure the electronic contact. The frequency range used was from 10^6^ to10^−2^ Hz, and the AC amplitude is 10 mV. The impedance was measured in humidified H_2_ (~3% H_2_O).

## Results and Discussion

### Electron Redistribution at Ni_10_-CeO_2_(111) by Computation

**Figure 1:**
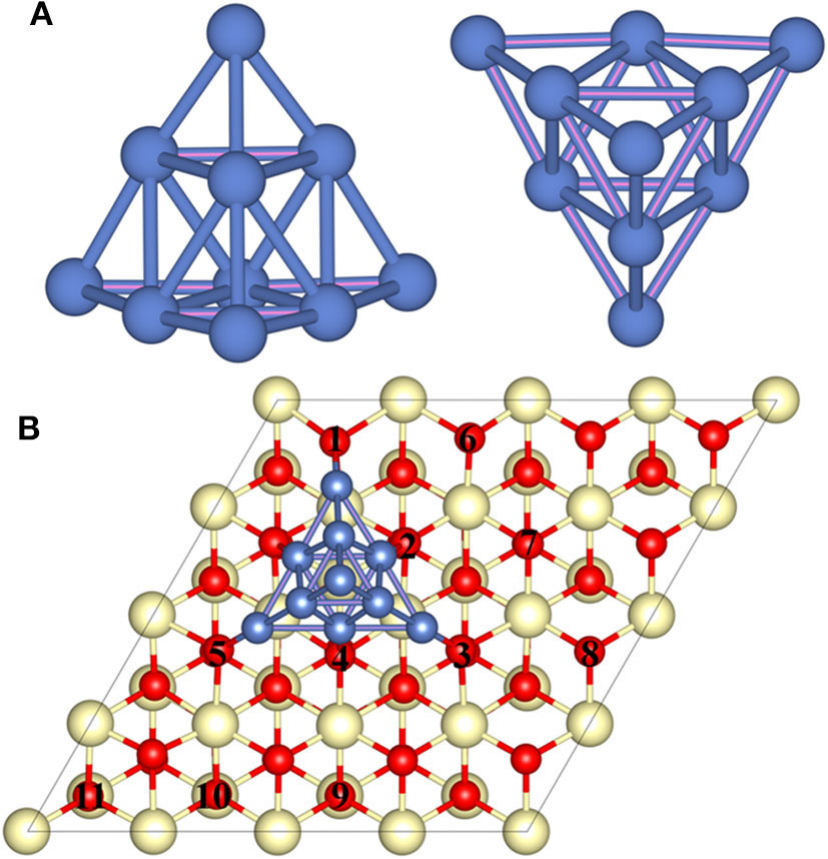
The side and top views of the optimized geometry structure of **(A)** the free-standing Ni_10_ cluster and **(B)** the optimized Ni_10_ cluster placed on the stoichiometric CeO_2_(111) with the lowest energy configuration. The dark blue, light yellow, and red balls refer to Ni, Ce, and O atoms, respectively. Numbers 1–5 refer to interface oxygen while 6–11 to surface oxygen.

A previous study has revealed that the trigonal pyramid-shaped Ni_10_ cluster with *T*_d_ symmetry is the most stable structure (Lu et al., 2011; Song et al., 2011; Rodríguez-Kessler and Rodríguez-Domínguez, 2015), and the low-index (111) surface of CeO_2_ is the most stable surface, so that CeO_2_(111) surface is selected as the substrate to represent CeO_2_ (Conesa, 1995). To simplify the calculation process, the effect of electrochemical environment such as potential is not considered in this work since the real reaction condition is too complicated to simulate precisely. Similar to previous studies of the metal–CeO_2_ systems, we focus on the effect of heterogeneous structure of the CeO_2_-supported metals, while the diffusion of metal particles is not considered (Kim and Henkelman, 2012; Kim et al., 2012; Kishimoto et al., 2014; Liu et al., 2016). Based on this, the pyramid-shaped Ni_10_ cluster is constructed on the CeO_2_(111) surface to illustrate the properties of Ni–CeO_2_ cermet for the H_2_ reaction process. Various positions for Ni_10_ cluster on CeO_2_(111) surface are investigated, and the configuration with the lowest energy is displayed in Figure 1, which will be used as the model to study the reaction process.

**Figure 2:**
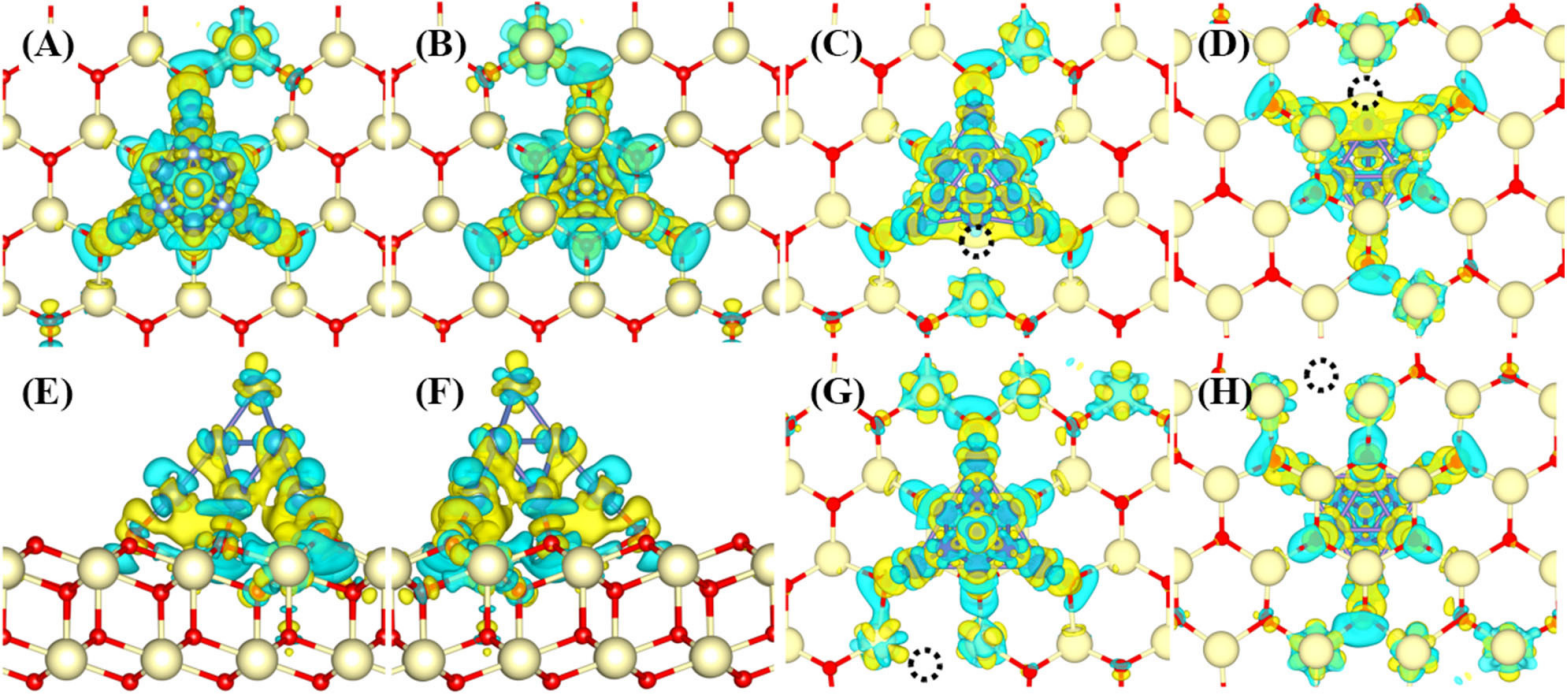
Charge density difference profiles for Ni_10_-CeO_2_(111) **(A–D)** without and **(E–H)** with oxygen vacancy. The views without vacancy are **(A)** from top to bottom, **(B)** from bottom to top, and **(C,D)** side. The views with an interface oxygen vacancy (V4) are **(E,F)** while **(G,H)** refer to surface oxygen vacancy (V10). The views **(E,G)** are from top to bottom while **(F,H)** from bottom to top. The dark blue, light yellow, red balls, and black dotted circle refer to Ni, Ce, O atoms, and oxygen vacancy, respectively. The isosurface value is 0.004 a.u. The yellow and blue colors refer to the positive and negative values, respectively.

The interaction between Ni_10_ cluster and CeO_2_(111) substrate is essential to elucidate the H_2_ oxidation pathways and electrochemical catalytic properties since the charge transfer between Ni_10_ and CeO_2_(111) determines the chemical activity of the substrate to a great extent. Bader charge analysis indicates that ~1.07 electrons are transferred from Ni_10_ cluster to CeO_2_(111) surface (Supplementary Figure 1). The redistributed charge density [i.e., the charge density difference between CeO_2_(111) with and without Ni_10_ cluster, is displayed in Figure 2]. It is found that the valence states of four Ce ions are reduced from Ce^4+^ to Ce^3+^ with three Ce ions under the Ni_10_ cluster and one adjacent to Ni_10_ cluster (Figures 2A–D). Such remarkable charge transfer from Ni_10_ cluster to CeO_2_(111) surface indicates that there is a strong interaction at the interface between Ni_10_ cluster and CeO_2_(111) surface, resulting in a partially positively charged Ni_10_ cluster on negatively charged CeO_2_(111) surface. Such structure also indicates that Ni can facilitate the formation of oxygen vacancy on the ceria surface.

It is known that oxygen defects may alter the electronic structure and thus influence the H_2_ oxidation reaction (Wu et al., 2015). Similar to previously reported configurations (Shishkin and Ziegler, 2010a,b), two types of oxygen vacancies are investigated (Supplementary Figure 2). The first type is the interface oxygen vacancy (labeled as V_4_), created by removing one oxygen atom at the interface of Ni_10_ and CeO_2_. The second type is the surface oxygen vacancy (labeled as V_10_), formed by removing one oxygen atom at the CeO_2_ surface away from Ni_10_. For model V_4_, the Bader charge analysis indicates that about 0.93 electrons are transferred from the Ni_10_ cluster to the CeO_2_ surface, which is smaller than that on the perfect CeO_2_(111) surface (1.07*e*). Although the presence of interface oxygen vacancy reduces the charge transferred from the Ni_10_ cluster to the CeO_2_ surface, still five Ce ions are found to be reduced to lower valences. As illustrated in Figures 2E,F, the transferred electrons are mainly located at two Ce ions neighboring to the Ni_10_ cluster, and the remaining three Ce ions are underneath the Ni_10_ cluster. However, for model V_10_, more charges are transferred from the Ni_10_ cluster to the reduced vacancy defect. The Bader charge analysis indicates that about 1.58 electrons are transferred from Ni_10_ cluster to the reduced CeO_2_(111) surface with surface oxygen vacancy (V_10_), which is significantly larger than those on the perfect and oxygen-defected CeO_2_(111) surface with an interface oxygen vacancy. The plotted deformation charge density profiles, illustrated in Figures 2G,H, show that the transferred electrons are mainly situated on five reduced Ce ions adjacent to the Ni_10_ cluster, and the reduced valence states of three Ce ions underneath the Ni_10_ cluster are negligible. Therefore, both surface and interface oxygen vacancy significantly enhance the reduction in valence state of Ce ions on the surface. Besides, the electronic state of cerium, whether it is at the interface or surface, is obviously affected by the presence of nickel cluster under both conditions of oxygen-nonstoichiometric and perfect crystalline. Accordingly, the electrochemical performance on the ceria surface can be quite different from that at the Ni–ceria interface.

### Computational Results of H_2_ Oxidation Reaction Pathway on Ni_10_-CeO_2_(111)

Since ceria itself is catalytically active to hydrogen oxidation, the reaction on the Ni–ceria system may proceed in two different situations, one on bare ceria and the other with involvement of Ni. The first pathway (pathway I) occurs on the bare ceria surface, which has been studied in our previous work (Wang et al., 2016). Briefly, pathway I starts with a relatively weak hydrogen adsorption on the oxide surface with the adsorption energy of −0.035 eV. H_2_ prefers to be adsorbed in parallel with the stoichiometric CeO_2_(111) surface, subsequently dissociating into two hydrogen atoms over surface oxygen atoms while forming two hydroxyl groups by overcoming an energy barrier of 1.073 eV. Finally, the two –OH species form an adsorbed water molecule and a residual oxygen vacancy with an energy barrier of 2.399 eV, which is the most energy-consuming step.

The reaction processes with the participation of Ni are energetically preferred yet more complicated than that on the bare ceria surface. The initial step of hydrogen adsorption may happen either on ceria or Ni. In the first scenario where hydrogen is adsorbed on ceria, the possible reaction route (pathway II) is followed by disassociation of hydrogen molecule at the Ni–ceria interface and H_2_O formation at 3PB as shown in the following steps A1–A4, where “sur” indicates ceria surface, and “inter” indicates the Ni–ceria interface.

$$\begin{array}{l} {\text{H}}_{2}\left(\text{g}\right)\to {\text{H}}_{2}^{*}\left(\text{ad},\text{sur}\right)\;\left(\text{A}1\right)\\ {\text{H}}_{2}^{*}\left(\text{ad},\text{sur}\right)\to {\text{H}}^{*}\left(\text{O}-\text{inter}\right)+{\text{H}}^{*}\left(\text{O}-\text{sur}\right)\;\left(\text{A}1-\text{A}2\right)\\ {\text{H}}^{*}\left(\text{O}-\text{inter}\right)+{\text{H}}^{*}\left(\text{O}-\text{sur}\right)\to {\text{H}}^{*}\left(\text{Ni}\right)-\text{O}{\text{H}}^{*}\\ \left({\text{V}}_{\text{O}}^{\cdot \cdot }-\text{sur}\right)\;\left(\text{A}2-\text{A}3\right)\\ {\text{H}}^{*}\left(\text{Ni}\right)-\text{O}{\text{H}}^{*}\left({\text{V}}_{\text{O}}^{\cdot \cdot }-\text{sur}\right)\to {\text{H}}_{2}\text{O}\left(\text{g}\right)+{\text{V}}_{\text{O}}^{\cdot \cdot }\left(\text{sur}\right)\;\left(\text{A}3-\text{A}4\right) \end{array}$$

**Figure 3:**
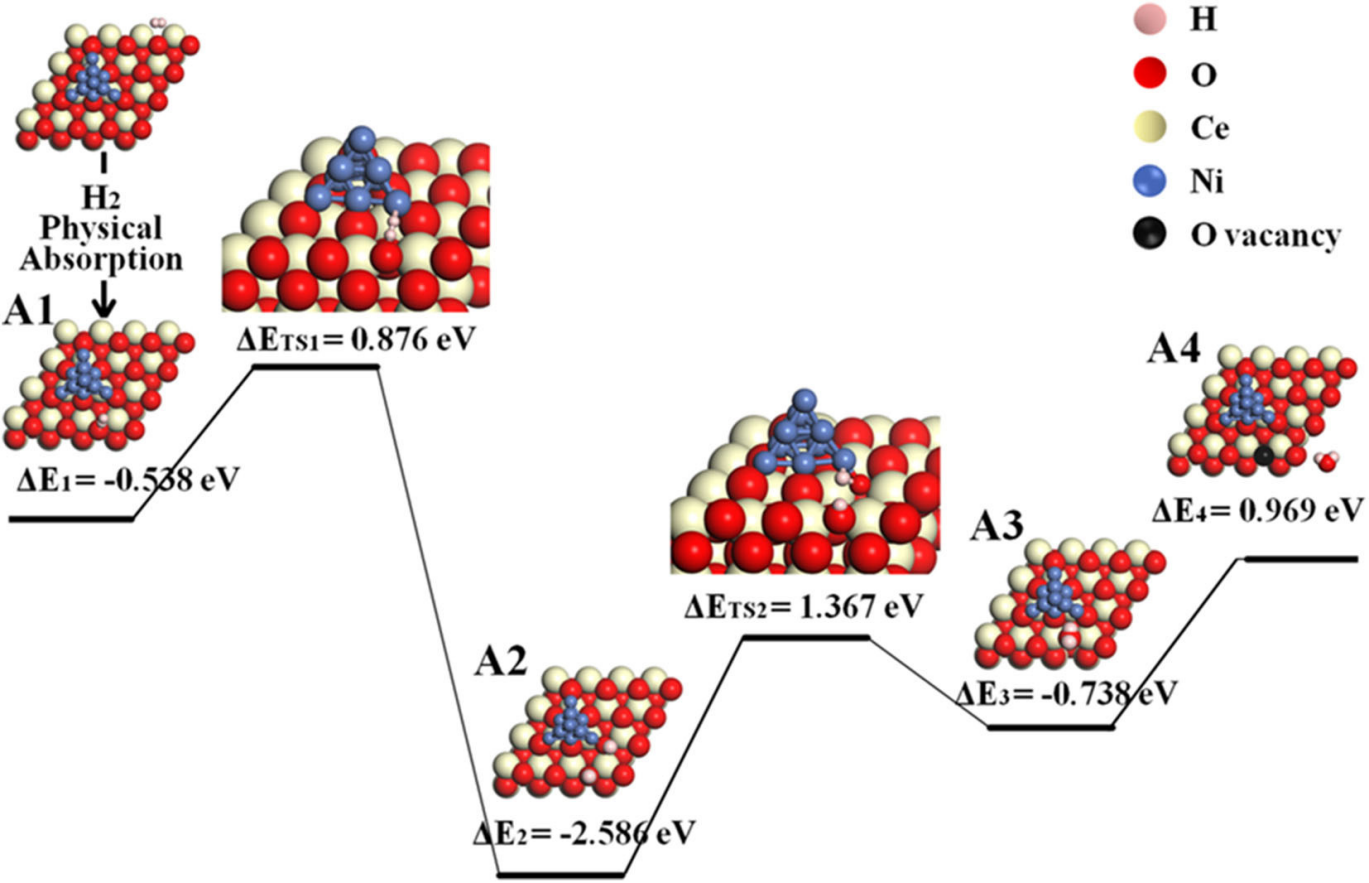
Molecular pathway of H_2_ oxidation reaction in Ni_10_-CeO_2_(111) system with hydrogen adsorption on ceria surface and oxidation reaction at 3PB (pathway II).

According to the calculated results displayed in Figure 3 for pathway II, the H_2_ molecule is physically adsorbed on the ceria surface away from Ni in the first step (A1), with an adsorption energy of −0.538 eV. The H–H bond length of the adsorbed H_2_ molecule is 0.754 Å (Supplementary Figure 3), slightly larger than the calculated equilibrium bond length of a free H_2_ molecule (0.740 Å) (Wang et al., 2016). Then, the adsorbed H_2_ dissociates into two separated hydrogen atoms: one adsorbed on interface oxygen, and the other adsorbed on the surface oxygen (A1–A2). This step includes an energy barrier of 0.876 eV, while the total energy difference from A1 to A2 is −1.710 eV, indicating that the formation of two –OH species is exothermic. In the next step, the two adsorbed –OH species combines to form an adsorbed H_2_O on Ni_10_ cluster by overcoming a high energy barrier of 1.367 eV (A2–A3). The formation of adsorbed H_2_O is endothermic with an energy difference of 0.629 eV. Finally, the adsorbed H_2_O molecule is released as gas phase by overcoming an energy barrier of 0.969 eV. In short, the whole molecular pathway gives off 0.650 eV, and the highest energy barrier of this exothermic reaction is 1.367 eV, which is much smaller than 2.399 eV for the H_2_ oxidation reaction on the bare CeO_2_(111) (Wang et al., 2016).

In the second scenario, hydrogen is adsorbed on nickel rather than ceria in the initial step. According to a previous research, Ni not only functions as electron acceptor but also catalyzes the dissociation of hydrogen molecule into atoms. The hydrogen atoms formed on the Ni surface may spill over to the surface of oxides such as zirconia (Anderson and Vayner, 2006; Shishkin and Ziegler, 2009), LnO_x_ (Ln = Dy, Ho, Er, Yb, and Tb) (He et al., 2011), and gadolinia-doped ceria (Babaei et al., 2009).

The possible H-spillover pathway involves hydrogen adsorption and disassociation on Ni, hydrogen spillover, and water formation. The proposed steps are summarized as B1–B7.

$$\begin{array}{l} {\text{H}}_{2}\left(\text{g}\right)\to {\text{H}}^{1*}\left(\text{Ni}\right)+{\text{H}}^{2*}\left(\text{Ni}\right)\;\left(\text{B}1/\text{B}2\right)\\ {\text{H}}^{1*}\left(\text{Ni}\right)+{\text{H}}^{2*}\left(\text{Ni}\right)\to {\text{H}}^{1*}\left(\text{O}-\text{inter}\right)\\ +{\text{H}}^{2*}\left(\text{Ni}\right)\;\left(\text{B}1-\text{B}3/\text{B}2-\text{B}4\right)\\ {\text{H}}^{1*}\left(\text{O}-\text{inter}\right)+{\text{H}}^{2*}\left(\text{Ni}\right)\to {\text{H}}^{1*}\left(\text{O}-\text{inter}\right)\\ +{\text{H}}^{2*}\left(\text{O}-\text{sur}\right)\;\left(\text{B}3-\text{B}5/\text{B}4-\text{B}5\right)\\ {\text{H}}^{1*}\left(\text{O}-\text{inter}\right)+{\text{H}}^{2*}\left(\text{O}-\text{sur}\right)\to {\text{H}}^{1*}\left(\text{O}-\text{inter}\right)\\ -\text{O}{\text{H}}^{2*}\left({\text{V}}_{\text{O}}^{\cdot \cdot }\right)\;\left(\text{B}5-\text{B}6\right)\\ {\text{H}}^{1*}\left(\text{O}-\text{inter}\right)-\text{O}{\text{H}}^{2*}\left({\text{V}}_{\text{O}}^{\cdot \cdot }\right)\to {\text{H}}_{2}\text{O}\left(\text{g}\right)+{\text{V}}_{\text{O}}^{\cdot \cdot }\;\left(\text{B}6-\text{B}7\right) \end{array}$$

**Figure 4:**
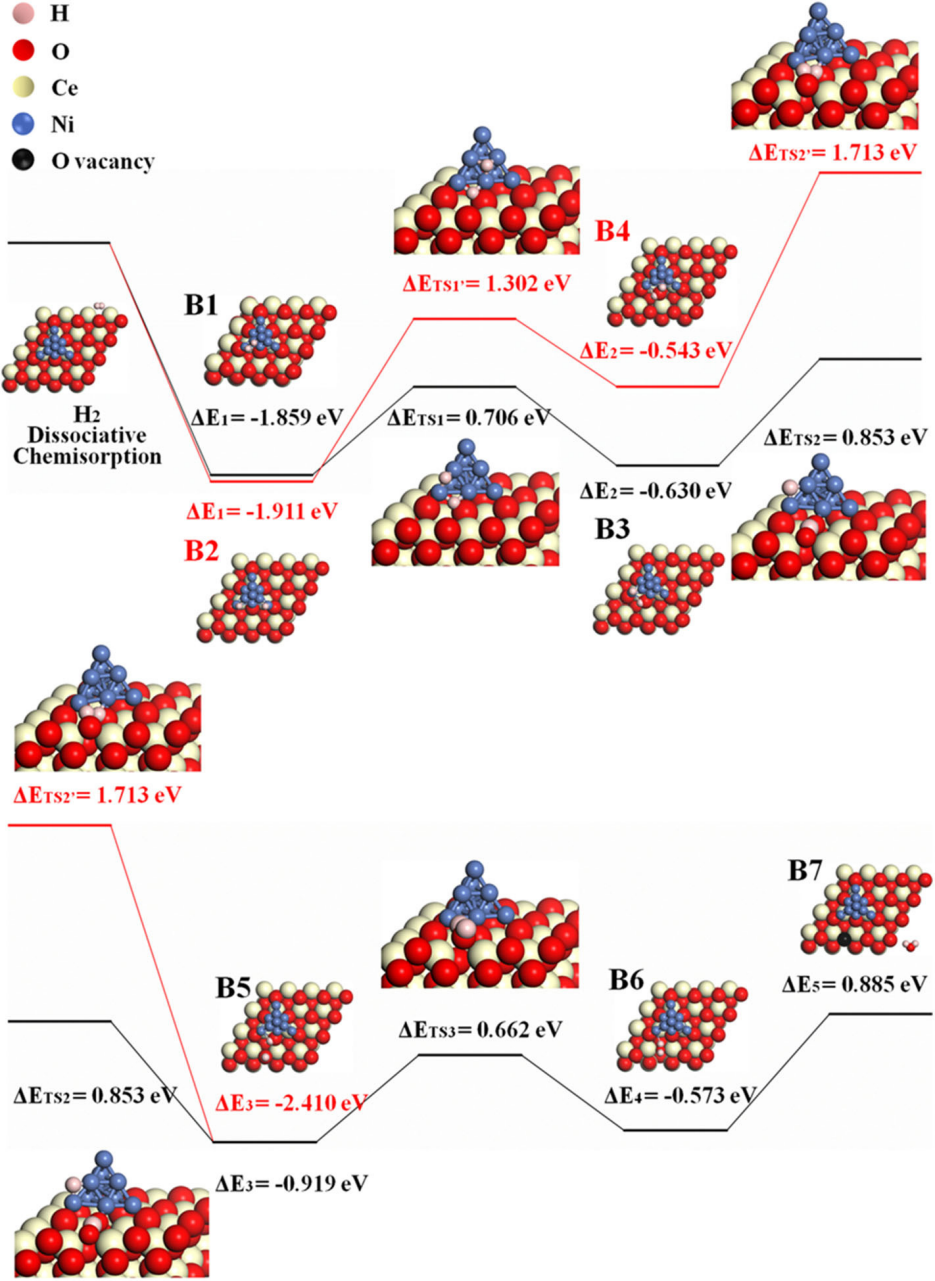
The H-spillover pathways of H_2_ oxidation in the Ni_10_-CeO_2_(111) system with hydrogen adsorption on Ni_10_ and oxidation reaction at 3PB (pathway III in black lines and pathway IV in red lines).

Because H_2_O may form either at the ceria surface or at 3PB of Ni–ceria–gas interface, two possible H-spillover pathways are distinguished as illustrated in Figure 4. Pathway III is labeled with black line, which proceeds with steps of B1, B3, and B5–B7, while red line refers to pathway IV that goes through B2, B4, and B5–B7. Both initial structures are dissociated H_2_ molecule on the Ni_10_ cluster surface (B1/B2). In pathway III, one dissociative H (H^1^) atom is adsorbed on the edge between the top and interlayer of Ni_10_ cluster, while the other H (H^2^) atom is adsorbed on the edge between the interlayer and contact layer of Ni_10_ cluster (B1). Then, H^1^ slightly moves and combines with an interface oxygen near the Ni_10_ cluster, while H^2^ spills over to the ceria surface away from the Ni_10_ cluster to combine with a surface oxygen. Thus, two hydroxyl groups are generated in this process, overcoming an energy barrier of 0.853 eV (B3–B5). After that, H^1^ is attracted to the hydroxyl group containing H^2^, forming one H_2_O molecule (B5–B6) and a surface oxygen vacancy (B6–B7) with energy barriers of 0.662 and 0.885 eV, respectively. In pathway IV, H atoms are adsorbed on two edges between the interlayer and the contact layer of Ni_10_ cluster in structure B2, which is slightly more stable than structure B1. In the following steps, H atoms migrate from the Ni_10_ cluster to the ceria surface and react with the oxygen atoms at interface and surface to form two –OH species, overcoming the energy barriers of 1.302 and 1.713 eV, respectively. In general, pathway III (black line) is more favorable compared to pathway IV (red line), since the highest energy barrier for pathway III (0.885 eV for B6–B7) is much lower than that for pathway IV (1.713 eV for B4–B5).

**Figure 5:**
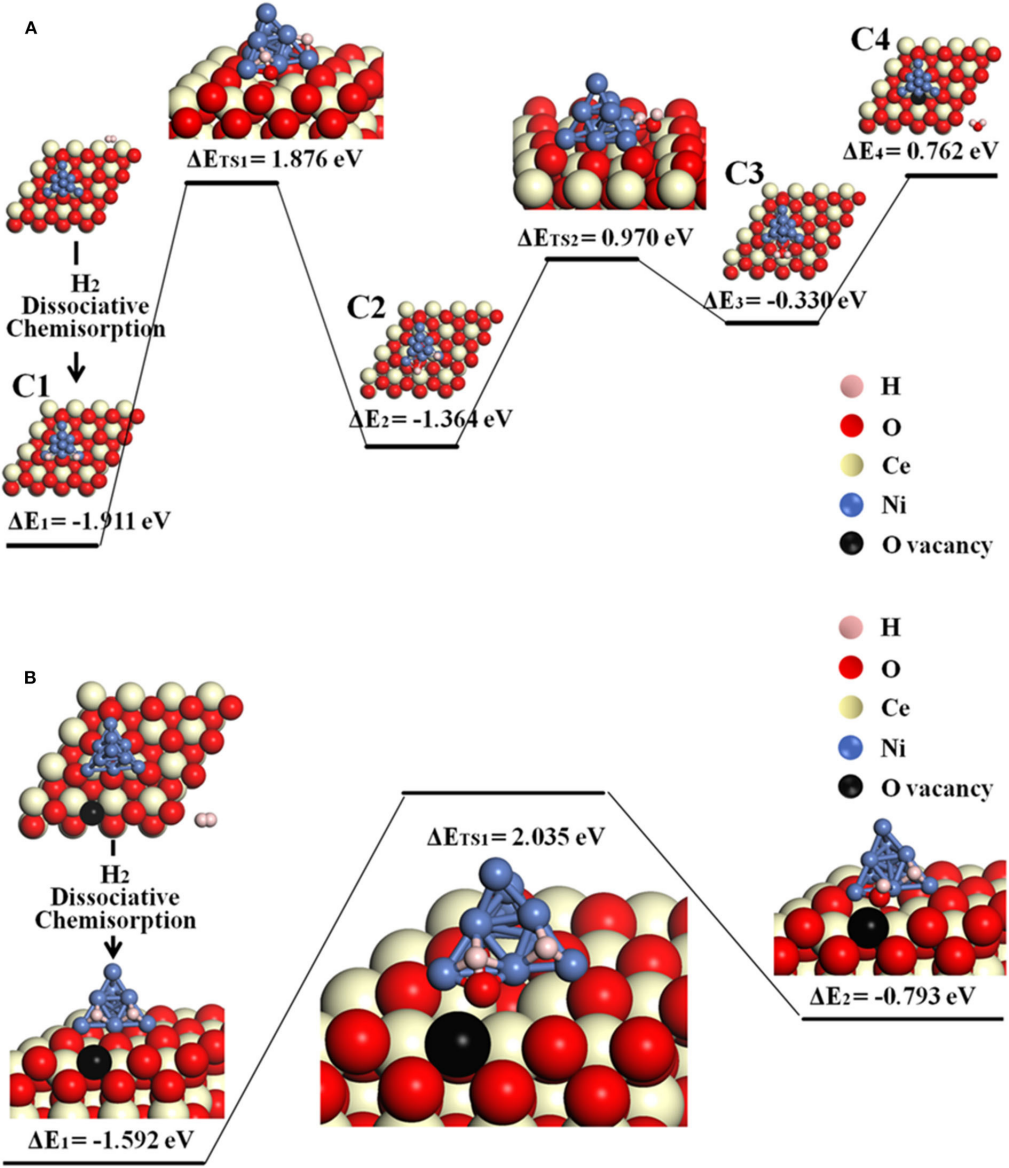
The O-spillover pathways of H_2_ oxidation in the Ni_10_-CeO_2_(111) system with hydrogen adsorption on Ni_10_ and oxidation reaction at 3PB: **(A)** pathway V for the stoichiometric structure and **(B)** pathway VI for nonstoichiometric structure with a surface oxygen vacancy.

The possible O-spillover pathways include hydrogen adsorption and dissociation on Ni, oxygen spillover from ceria to Ni cluster, and formation of H_2_O molecule, summarized as C1–C4 and illustrated in Figure 5A for pathway V.

$$\begin{array}{l} {\text{H}}_{2}\left(\text{g}\right)\to {\text{H}}^{1*}\left(\text{Ni}\right)+{\text{H}}^{2*}\left(\text{Ni}\right)\;\left(\text{C}1\right)\\ \text{O}\left(\text{inter}\right)\to {\text{O}}^{*}\left(\text{Ni}\right)+{\text{V}}_{\text{O}}^{\cdot \cdot }\left(\text{inter}\right),{\text{O}}^{*}\left(\text{Ni}\right)\\ +{\text{H}}^{1*}\left(\text{Ni}\right)\to \text{O}{\text{H}}^{1*}\left(\text{Ni}\right)\;\left(\text{C}2\right)\\ {\text{H}}^{2*}\left(\text{Ni}\right)+\text{O}{\text{H}}^{1*}\left(\text{Ni}\right)\to {\text{H}}_{2}\text{O}\left(\text{Ni}\right)\;\left(\text{C}2-\text{C}3\right)\\ {\text{H}}_{2}\text{O}\left(\text{Ni}\right)\to {\text{H}}_{2}\text{O}\left(\text{g}\right)\;\left(\text{C}3-\text{C}4\right) \end{array}$$

The initial structure C1 is for the disassociation of hydrogen molecule. In the following step, an oxygen atom at the Ni_10_-CeO_2_ interface spill overs from the oxide surface toward the metal cluster, then reacts with the hydrogen atom to form a hydroxyl group while leaving an interface oxygen vacancy (C1–C2). This is the most energy-consuming step, as it contains the highest energy barrier of 1.876 eV. Next, the hydroxyl group reacts with H atom, forming an adsorbed H_2_O on the Ni_10_ cluster by overcoming an energy barrier of 0.970 eV (C2–C3). In the final step, it consumes 0.762 eV to release the adsorbed H_2_O molecule to the gas phase. As a whole, this O-spillover process is endothermic with a negligible energy of 0.003 eV, and the rate-limiting process is H_2_ oxidation.

Besides, the situation where an oxygen vacancy is created on the ceria surface is also considered, as illustrated in Figure 5B for pathway VI. In this case, the adsorption energy for the first step (H_2_ disassociation on Ni_10_ cluster) is −1.592 eV, which is less stable than the C1 structure. Next, interface O atom migrates to the Ni surface and reacts with one H atom to form an –OH species by overcoming a large energy barrier of 2.035 eV. Doubtlessly, it takes more energy to achieve a O-spillover step when a surface oxygen vacancy exists on the ceria.

In conclusion, six possible pathways are proposed for the oxidation reaction on the Ni–ceria system. Pathway I takes place on the bare ceria surface and is entirely unrelated to the Ni–ceria interface. The other five pathways are all 3PB related since the reaction steps are either linking directly to the interface oxygen atoms (positions 1–5 in Figure 1) or to the surface oxygen atoms (positions 6–11 in Figure 1), noting that surface atoms are also affected by the nickel cluster as mentioned in the previous discussion. In addition, the surface atoms in our Ni–CeO_2_(111) model at the atomic scale could be regarded as the interface atoms in real electrochemical system, since the electrode is often characterized in a much larger scale such as nanoscale. Thus, it is reasonable to treat pathways II–VI as the possible reaction processes for the hydrogen oxidation reaction at the Ni–ceria interface while only pathway I as the pathway on the ceria surface.

Table 1 lists the hydrogen adsorption energy, highest energy barrier, and total energy balance of each pathway. The hydrogen adsorption energy differs enormously depending on the location. The energy for adsorption on bare ceria in pathway I is only −0.035 eV, while the adsorption energy in the Ni_10_ cluster in pathways III, IV, and V are around −1.9 eV. Besides, the adjacent Ni_10_ cluster in pathway II promptly increases the adsorption energy on the surface site, from −0.035 to −0.538 eV. Thus, the enhancement of hydrogen adsorption by Ni particles is quite obvious. Among the five pathways related to the Ni–ceria interface, the molecular pathway (pathway II) and the two H-spillover pathways (pathways III and IV) are all finished with formation of a surface oxygen vacancy rather than interface oxygen vacancy. Energetically speaking, pathway III is more favored than pathways II and IV. The highest energy barrier in pathway III is only 0.885 eV, about two-thirds of pathway II and one-half of pathway IV, while the energy balance is the lowest, −0.875 eV. In contrast, the highest energy barriers in pathways V and VI proceeding with oxygen spillover are more than twice as large as that in pathway III. Besides, the energy balance of pathway V (0.003 eV) is contradictory to the thermodynamic prediction. Consequently, the oxygen spillover pathways rarely contribute to the overall anodic reaction, as they are unlikely to occur.

**Table 1:** Adsorption energy and highest energy barrier for H_2_ oxidation reaction at Ni_10_-CeO_2_ (111) *via* various pathways.

	**Adsorption energy (eV)**	**Highest energy barrier (eV)**	**Energy Balance (eV)**
Pathway I, adsorption and reaction at bare ceria [Wang et al., 2016].	−0.035	2.399	−0.178
Pathway II, adsorption on ceria and reaction at interface	−0.538	1.367	−0.650
Pathway III, adsorption on Ni, hydrogen spillover, and reation at 3PB	−1.859	0.885	−0.875
Pathway IV, adsorption on Ni, hydrogen spillover, and reation at 3PB	−1.911	1.713	−0.875
Pathway V, adsorption on Ni, oxygen spillover, and reation at 3PB	−1.911	1.876	0.003
Pathway VI, adsorption on Ni, oxygen spillover, and reation at 3PB with surface oxygen vacancy	−1.592	2.035	−0.350

It is noted that the mechanism of hydrogen oxidation for Ni–CeO_2_ system is quite different from that for Ni–YSZ system, although both are controlled by the reaction at 3PB. For Ni–YSZ, the energetically favored pathway is hydrogen spillover from Ni to the Ni–YSZ interface with formation of an interface vacancy, while hydrogen spillover from the interface to YSZ surface with formation of a surface vacancy is very unfavorable because interfacial vacancy formation is much easier than the latter (Shishkin and Ziegler, 2014). However, due to facile formation of the surface vacancy at the CeO_2_(111) surface, the hydrogen oxidation for the Ni–CeO_2_ system tends to proceed with hydrogen spillover to the CeO_2_(111) surface and finish with the formation of a surface oxygen vacancy. This result is obvious for the energetically most favored route (pathway III).

As a result, assumption could be made that the reaction rate for interface process that mainly occurs following pathway III should be much higher than that for the ceria surface of pathway I, although both of them could exist in practical situations. Besides, the contribution from the surface reaction to the total electrode reaction could be very small, and the total reaction rate may be controlled by the interface length (i.e., the length of 3PB). The surface and interface reaction rates and their contributions will be further compared in *Experimental*, with the perspective of reaction kinetics.

### Experimental Results and Discussion

The hydrogen oxidation reaction catalyzed by ceria can be represented using Kroger–Vink notations as

(2) $$\begin{array}{r} H_{2}\left(g\right)+O_{O}^{\times }\;+2Ce_{Ce}^{\times }(Ce^{+4})\to H_{2}O\left(g\right)+V_{O}^{\cdot \cdot }+2Ce_{Ce}{}^{\prime }(Ce^{+3}) \end{array}$$

Equation (2) can also be considered as the ceria reduction reaction. Comparing Equations (1, 2), it is found that the ceria reduction reaction is equivalent to the anodic H_2_ oxidation reaction (Wang et al., 2012). Thus, ECR method is adopted to simulate the anodic reaction. By increasing the H_2_ partial pressure, the equilibrium in Equation (2) shifts toward the right. Meanwhile, Ce^4+^ is reduced to Ce^3+^, and the conductivity increases. The conductivity change is tested with dense bar samples.

**Figure 6:**
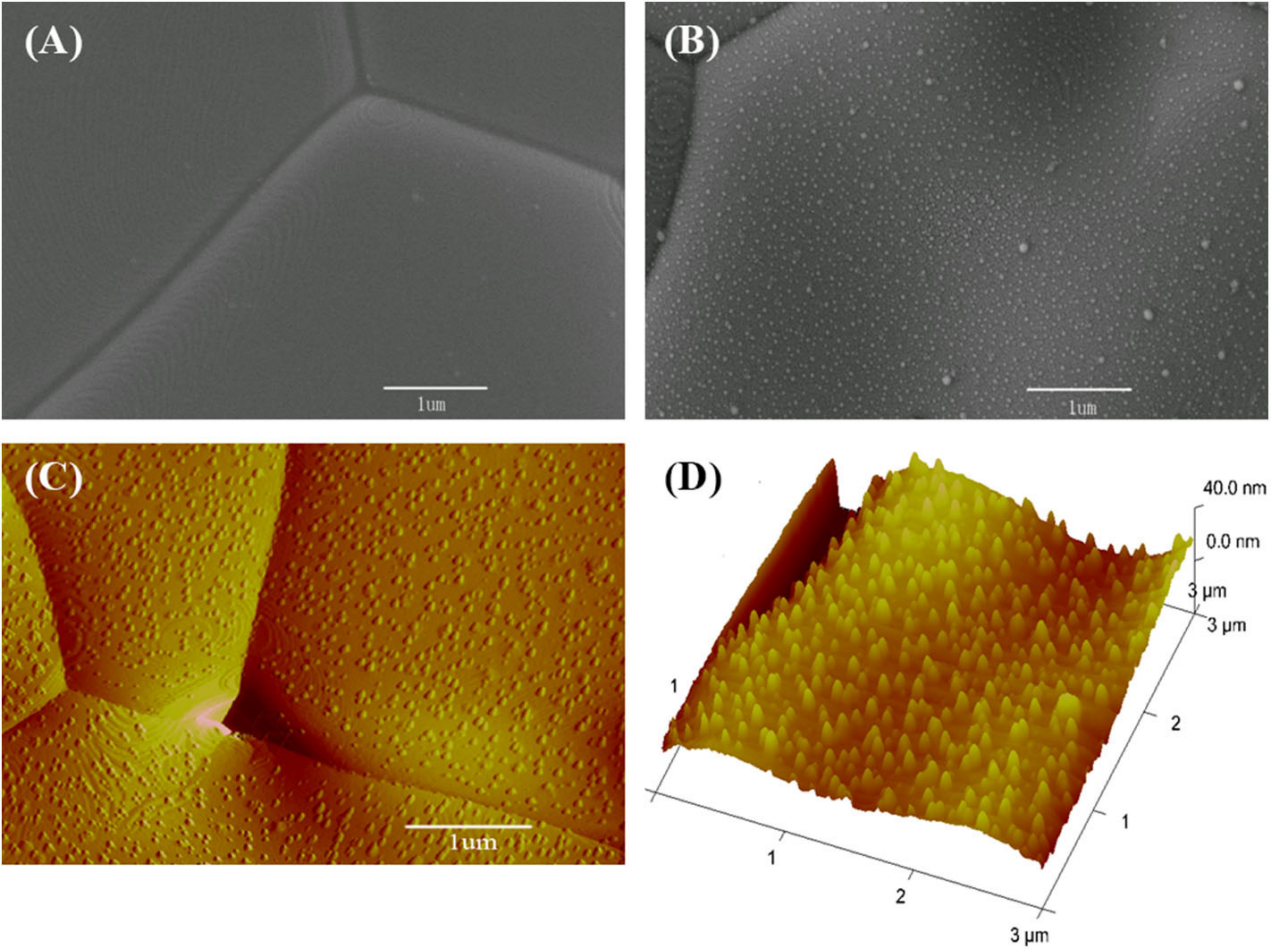
Surface micrographs for **(A)** scanning electron microscopy (SEM) view of a bare CeO_2_ bar, **(B)** SEM, **(C)** atomic force microscopy (AFM), and **(D)** three-dimensional AFM pictures for the bar with Ni particles prepared by 40 s deposition.

The CeO_2_ bar consists of grains several micrometers in size (Figure 6A). When Ni is deposited, the surface clearly presents fine particles (Figure 6B). These fine particles are Ni as formed in the sputtering–heating process, in which the sputtering results in a thin nickel film while the heating turns the film into particles. The fine particles are isolated and evenly distributed. The non-connected Ni particles do not influence the ground conductivity of the ceria bar. The Ni particle number per unit area increases with the sputtering time (Supplementary Figure 4). The diameter and vertical height of Ni particles are about 20 and 13 nm, respectively, as shown from the three-dimensional AFM pictures (Figures 6C,D).

**Figure 7:**
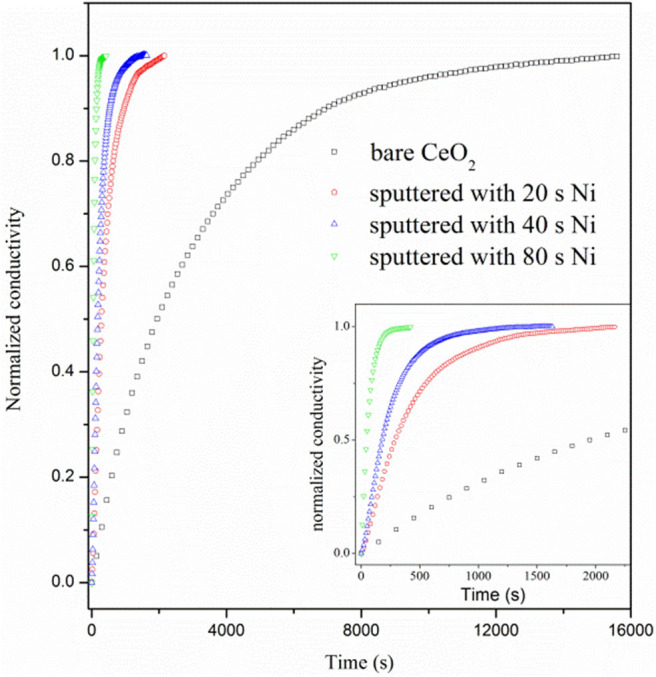
Normalized conductivity curves vs. relaxation time for bare CeO_2_ and CeO_2_ with Ni particles, which are prepared by 20, 40, and 80 s deposition.

Figure 7 shows the curves of normalized conductivity measured at 800°C. The surface H_2_ oxidation reaction rate for the bare CeO_2_ is very slow as the re-equilibrium time is over 12,000 s, which is much longer than that of doped ceria, about 7,000 s (Wang et al., 2013). When Ni particles are deposited on the ceria surface, however, the re-equilibrium time is sharply reduced. Besides, the re-equilibrium appears to be faster for the sample with longer deposition time of Ni. The re-equilibrium times for samples with 20, 40, and 80 s deposition are 2,171, 1,520, and 426 s, respectively. The accelerated re-equilibrium demonstrates enhanced surface reaction kinetics caused by Ni particles. The process of conductivity relaxation consists of the surface reaction to form oxygen ion vacancy and bulk diffusion of oxygen ions. Since the reaction is dominated by the surface process, the ECR data can be fitted with following formula (Yasuda and Hikita, 1994; Wang et al., 2013).

(3) $$\begin{array}{r} \frac{\sigma \left(t\right)-\sigma \left(0\right)}{\sigma \left(\infty \right)-\sigma \left(0\right)}=1-\exp\left(-\frac{k_{chem}t}{a}\right) \end{array}$$

where *k*_*chem*_ is the chemical surface exchange coefficient, which quantitatively represents the reaction rate of Equation (1), and the fitted *k*_*chem*_ values are listed in Supplementary Table 1. σ(0) and σ(∞) are the equilibrated conductivities at the initial and final time of the relaxation curve. *a* is a constant and numerically equals half of the bar sample thickness. *k*_chem_ is 0.72 × 10^−5^ cm s^−1^ for the bare CeO_2_ at 800°C, which equals to the reaction rate constant for hydrogen reduction on ceria surface, *k*_ceria_. When Ni sputter is conducted for 20, 40, and 80 s, the *k*_chem_ dramatically increases to 6.03 × 10^−5^, 10.3 × 10^−5^, and 39.1 × 10^−5^ cm s^−1^, respectively, proving that the surface reaction rate is remarkably accelerated by the involvement of Ni particles.

Physically, *k*_chem_ is contributed by the reaction on the bare ceria surface and the reaction related to the Ni particles, *k*_Ni_ (Wu et al., 2015)

(4) $$\begin{array}{r} k_{Ni}=k_{chem}-K_{ceria}\left(1-\theta \right) \end{array}$$

**Figure 8:**
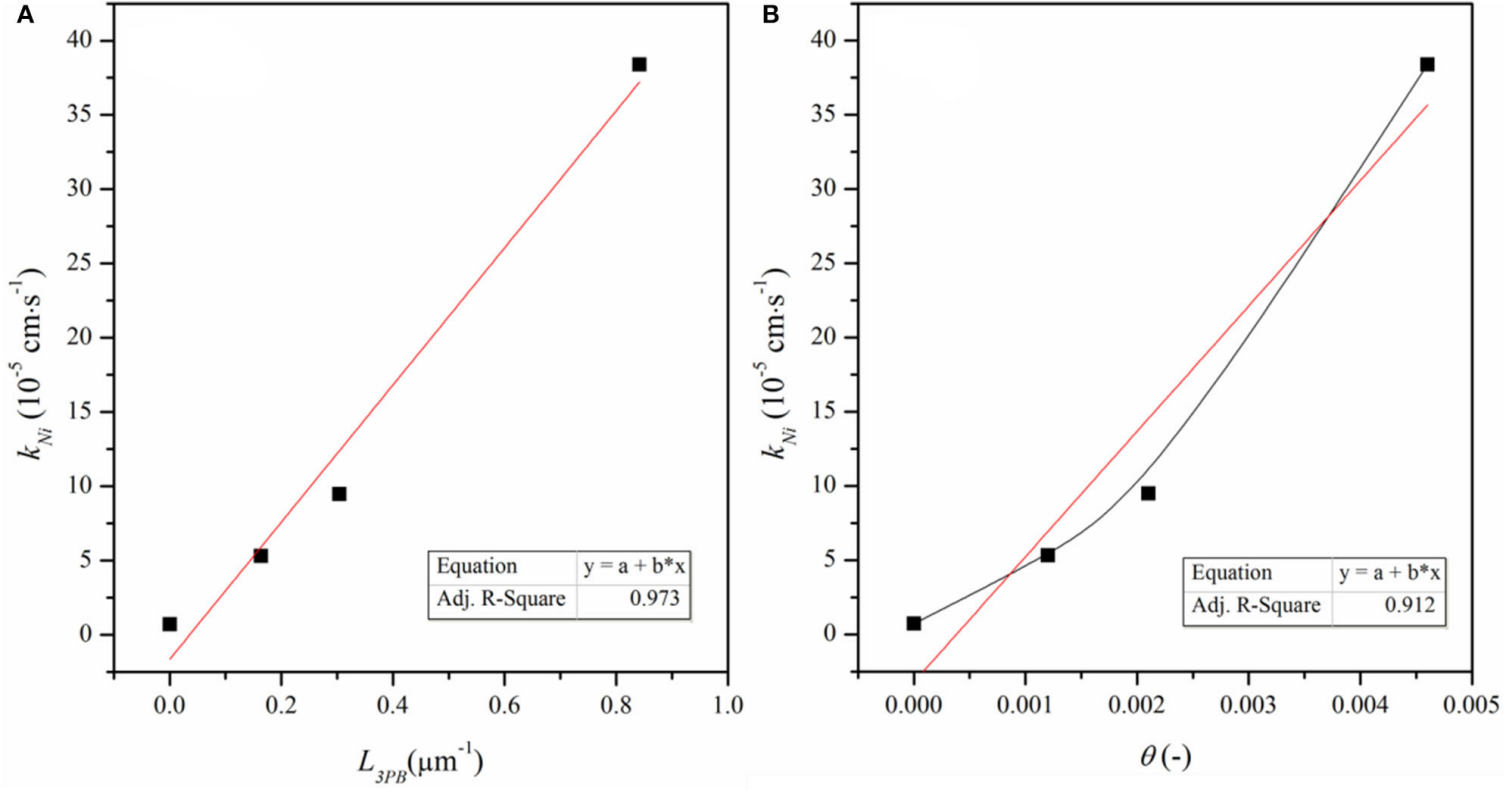
*k*_*Ni*_ as a function of **(A)** density of 3PB (*L*_3PB_) and **(B)** surface coverage of Ni particles, (θ).

*k*_Ni_ represents the nickel effect on the reaction, θ is the surface coverage of the Ni particles and (1 – θ) is surface fraction of the bare ceria (Supplementary Table 1). Figure 8 shows *k*_*Ni*_ as a function of θ and *L*_3PB_, the length of Ni–CeO_2_ interface (i.e., the length of 3PB boundaries). *k*_*Ni*_ increases linearly with *L*_3PB_, suggesting that *k*_*Ni*_ represents the reaction that is limited by the step occurring at/near the Ni–CeO_2_ interface rather than on the Ni–gas surface. According to the DFT analysis, the hydrogen reduction reactions are mostly favored by hydrogen-spillover-based pathway III, which is also limited to the Ni–CeO_2_-gas 3PB. Thus, the DFT approach agrees well with the ECR conclusion that the Ni-related reaction mainly occurs at/near the Ni–CeO_2_-gas 3PB.

According to the discussion based on DFT calculation, both reactions at the Ni-ceria interface and on the ceria surface may contribute to the H_2_ oxidation. Under the ECR experimental conditions, the reaction rate constant *k*_*Ni*_ for H_2_ oxidation at the interface is much higher than that on the ceria surface *k*_*ceria*_ (Supplementary Table 1). In addition, $\frac{k_{Ni}}{k_{ceria}}$ increases with *L*_3PB_ since a higher 3PB density means more active sites for the catalytic reaction. The highest $\frac{k_{Ni}}{k_{ceria}}$ value is about 53, demonstrating that the interface reaction is dominant although surface reaction exists. The ECR derived faster reaction rate at the interface than on the surface and is well-supported by the lower energy barrier in pathway III than pathway I as obtained by the DFT calculation.

**Figure 9:**
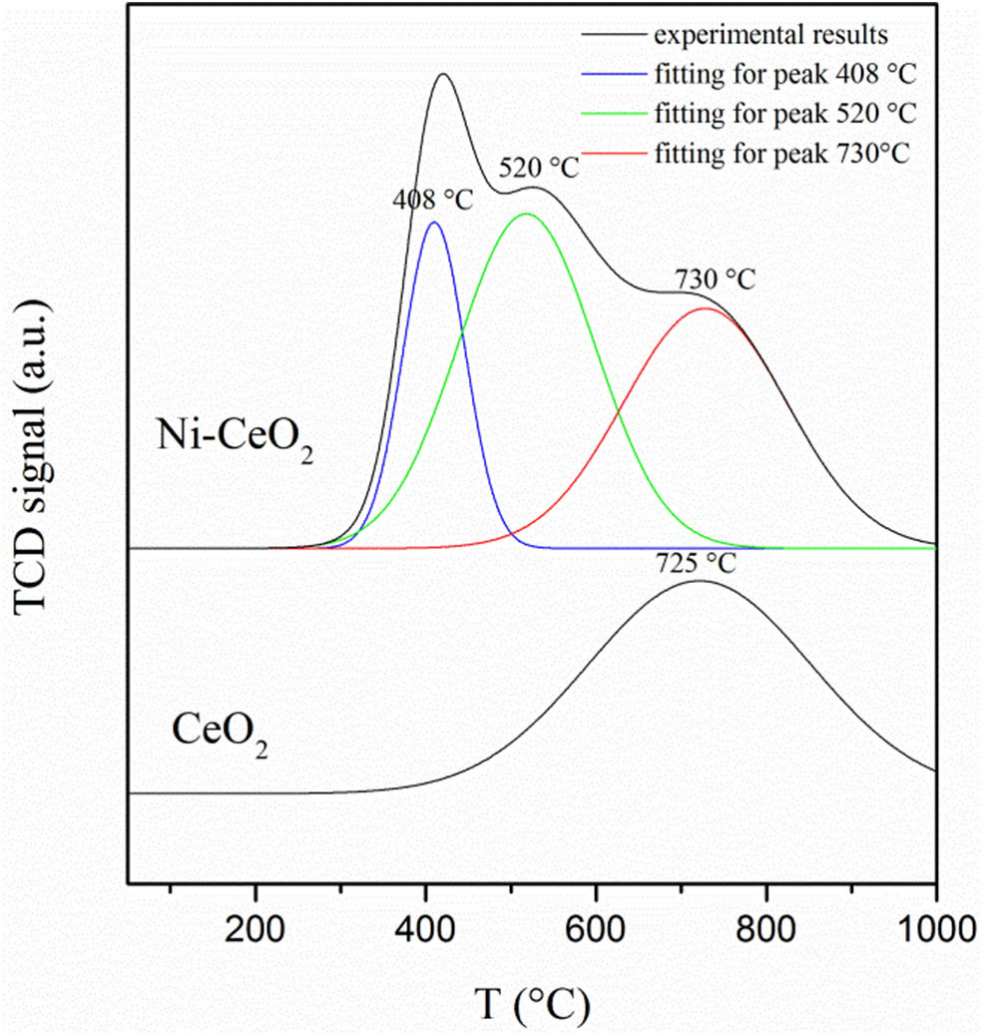
Hydrogen temperature programmed desorption profiles for CeO_2_ and Ni–CeO_2_ powders.

The hydrogen adsorption/desorption on the ceria surface and Ni–ceria interface is compared with hydrogen-TPD measurement (Figure 9). The desorption curve for bare ceria shows only one desorption peak, which shows that the hydrogen desorption/adsorption on the ceria surface begins at about 450°C and reaches its highest rate at 725°C. Quite differently, the curve for the Ni-ceria includes two additional peaks at 408 and 520°C. These two peaks must be attributed to the adsorption on Ni particles. The additional adsorption on Ni as demonstrated with TPD analysis is well-consistent with the high adsorption energy for Ni_10_-CeO_2_(111) obtained from DFT calculation.

**Figure 10:**
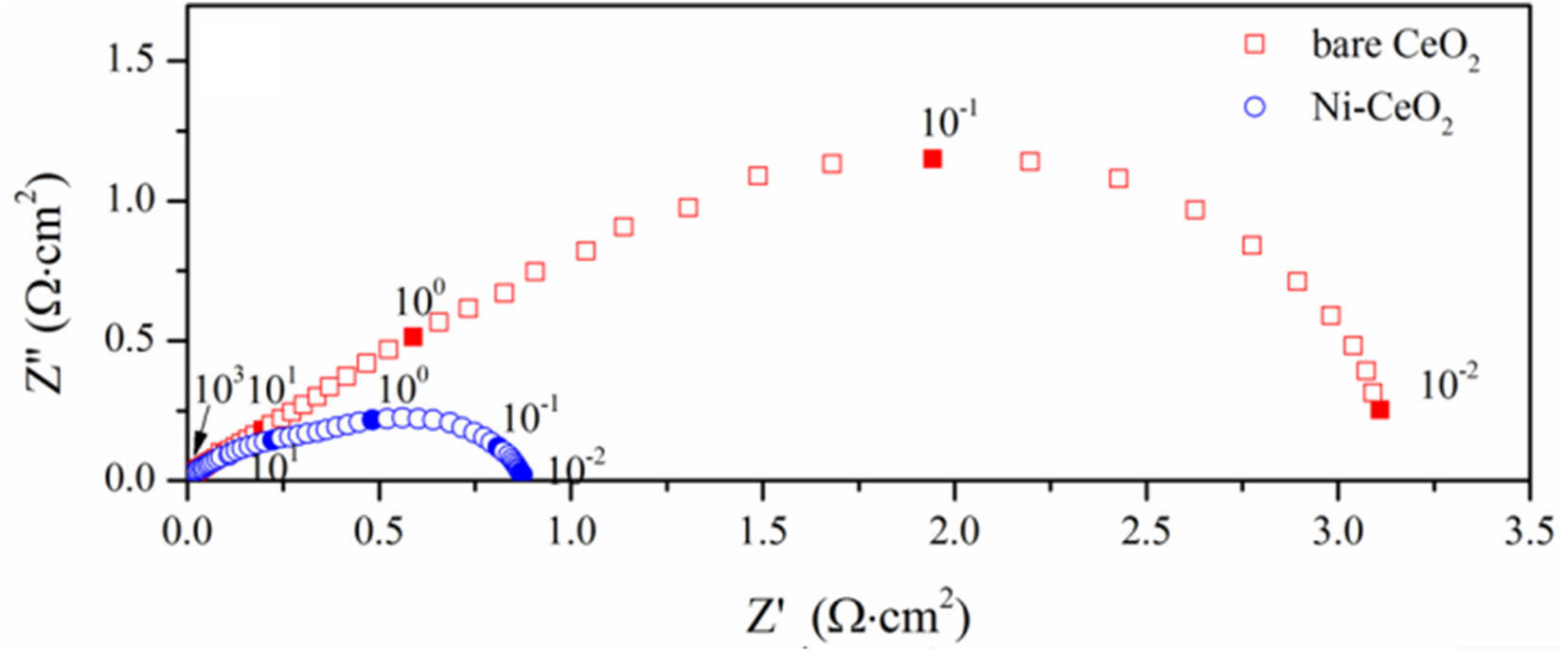
Impedance spectra measured at 800°C.

The electrochemical performance is compared with symmetrical cell using YSZ as the electrolyte substrates. Figure 10 shows the impedance spectra measured at 800°C, where the response corresponding to the ohmic resistance is deducted to compare the electrode response clearly. The interfacial polarization resistance is 3.18 and 0.88 Ω cm^2^ for the ceria and Ni-ceria electrodes, respectively. The reaction at the porous ceria electrode takes place at the ceria surface, while the Ni-ceria electrode provides additional reaction sites at the Ni-ceria interface. Since only 5.5% Ni is infiltrated to porous ceria to form the Ni-ceria electrode, the difference in electrode polarization resistance could roughly be attributed to the interface considering that the two electrodes have almost the same electronic conductivities and pore structures. Thus, the presence of the Ni-ceria interface in this work improves the electrochemical performance by 72.3%, proving that 3PB dominates the electrode reaction on Ni-ceria cermet anodes, which is possibly associated with the improved hydrogen adsorption capability and reduced energy barrier for hydrogen reduction.

## Conclusion

Both theoretical calculations and experiments are conducted regarding Ni–CeO_2_ as anode material for SOFCs. Via DFT + U computations, charge transfer between Ni_10_ cluster and CeO_2_(111) surface proves the synergistic effects existing in the Ni-ceria system. Then, five possible hydrogen oxidation pathways over the Ni–CeO_2_ electrode are proposed, including different sites for adsorption and reaction to evaluate the effects of Ni on CeO_2_(111) surface. The results demonstrate that nickel can largely enhance H_2_ adsorption on the ceria surface and lowers the energy barrier for disassociated hydrogens to combine with a surface oxygen of CeO_2_ and form H_2_O. The highest energy barrier decreases from 2.399 eV on pure CeO_2_ to 0.885 eV at the Ni–CeO_2_ interface in pathway III with hydrogen spillover mechanism, which can be concluded as the most energetically favored pathway. Furthermore, experimental results are found to be consistent with the theoretical calculations. ECR experiments demonstrate that surface reaction rate *k*_Ni_ increases linearly with the density of 3PB, and 98% of the total hydrogen oxidation is contributed by interface reaction at 3PB. H_2_-TPD measurements also show enhanced hydrogen adsorption caused by Ni, as two additional and stronger desorption peaks appear at lower temperatures compared to bare ceria. Finally, EIS of ceria and Ni-ceria electrodes are measured to compare the electrochemical performance. The interfacial polarization resistance is remarkably reduced from 3.18 Ω cm^2^ for pure ceria electrode to 0.88 Ω cm^2^ for Ni-ceria electrode at 800°C.

## Data Availability Statement

The original contributions presented in the study are included in the article/Supplementary Materials, further inquiries can be directed to the corresponding author/s.

## Author Contributions

CX and XW conceived of the idea, designed the calculations and experiments, and analyzed the data. YJ and MZ carried out the electrochemical experiments. JX conducted the characterizations. SW performed the theoretical calculations. YY participated in the discussion of the results. YJ and SW wrote the paper. All authors commented on the manuscript.

## Conflict of Interest

The authors declare that this study received funding from the National Natural Science Foundation of China (51972298) and Anhui Estone Materials Technology Co., Ltd. (2016340022003195). The funders were not involved in the study design, collection, analysis, interpretation of data, the writing of this article, or the decision to submit it for publication.
