# Supplementary material for: Hydrogen Oxidation Pathway Over Ni–Ceria Electrode: Combined Study of DFT and Experiment
Source: Front Chem. 2021 Feb 1;8:591322. doi: 10.3389/fchem.2020.591322 (PMC7882610; doi:10.3389/fchem.2020.591322)
Supplement: Supplementary file 1 [file Table_1.DOCX]

***Supplementary information***


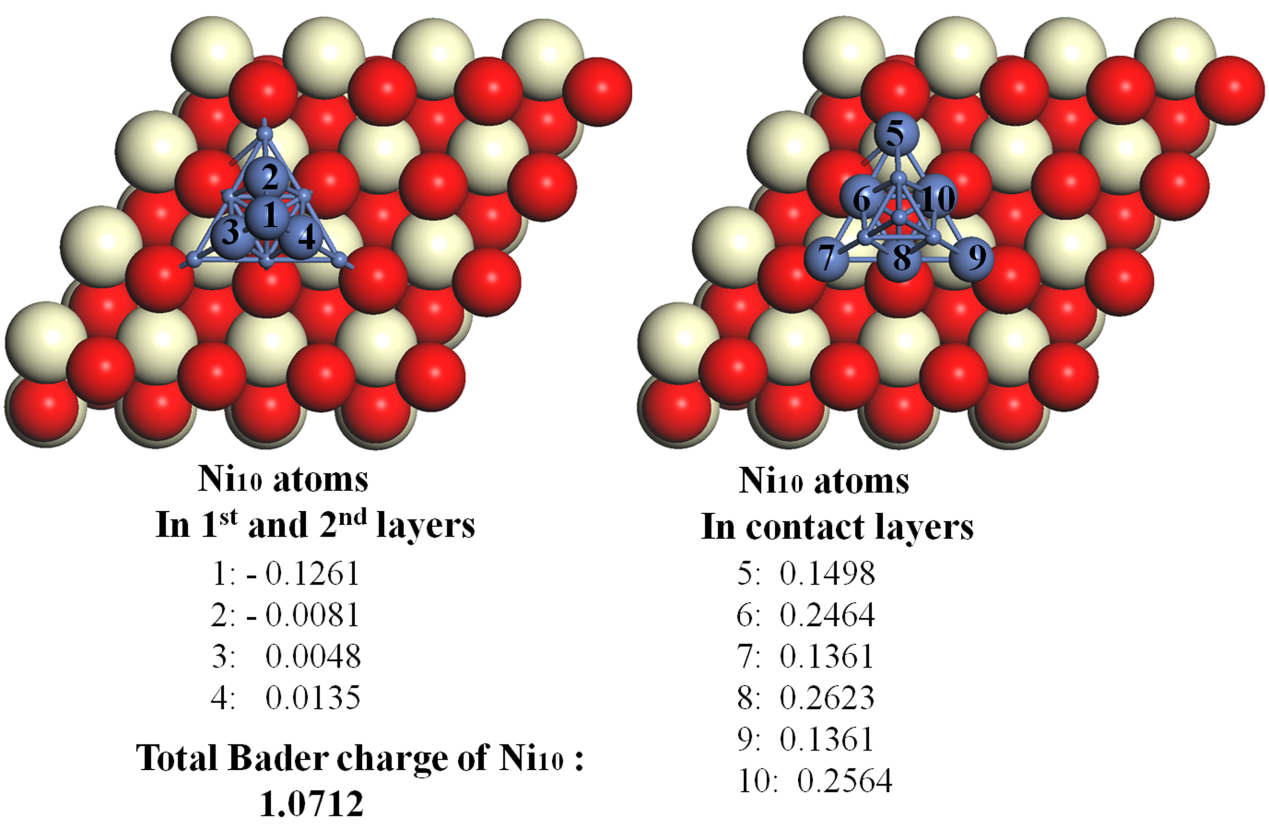


**Figure S1** Bader charge analysis on Ni_10_-CeO_2_(111) is shown with the quantity of electronic charge of every Ni atom.


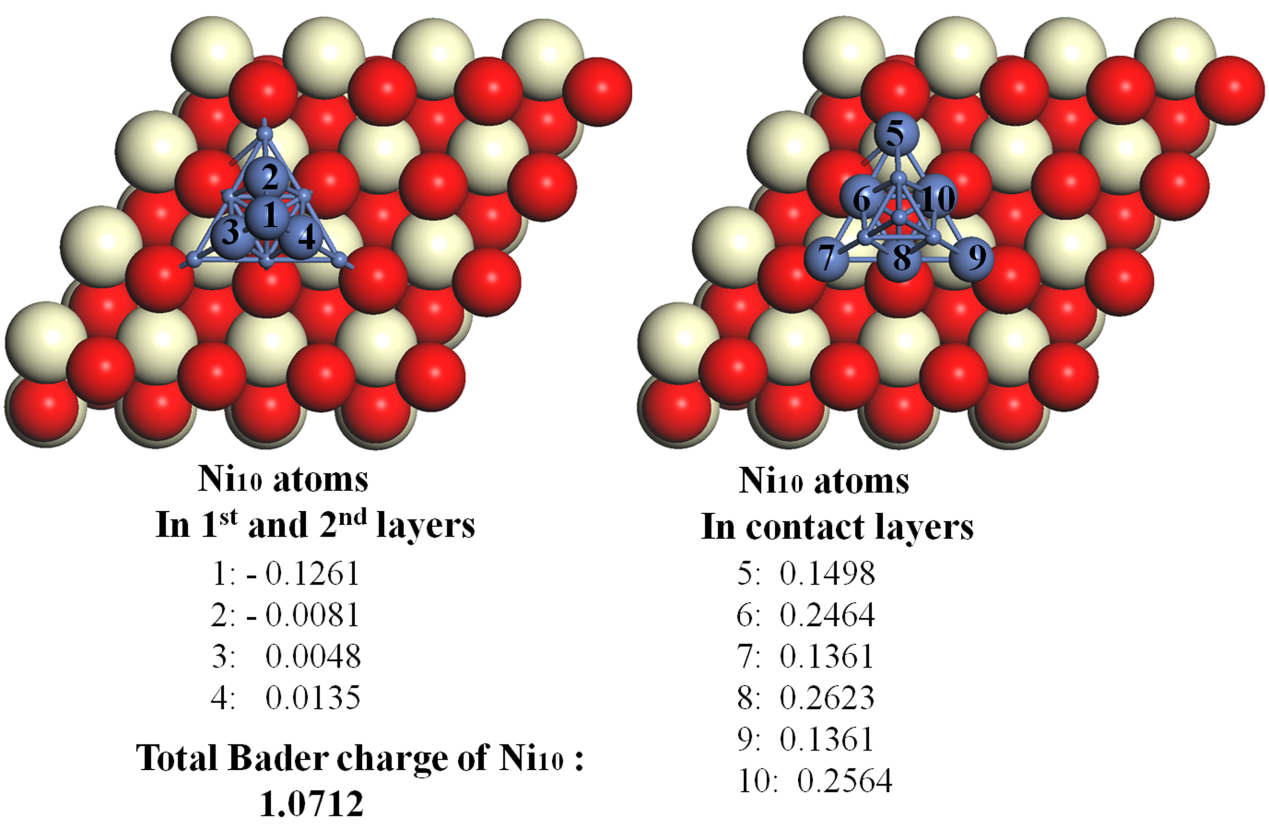

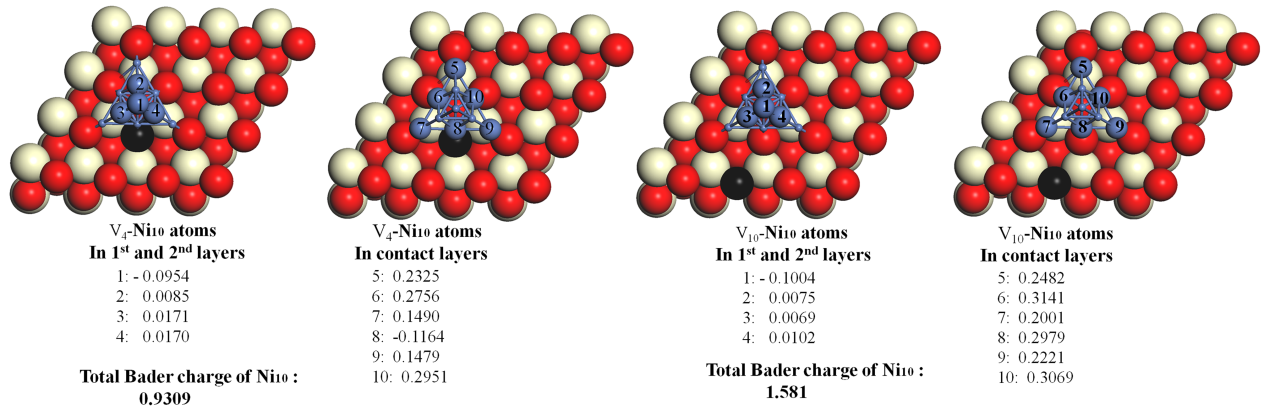


**Figure S2** Bader charge analysis of the Ni_10_-CeO_2_(111) system with two kinds of oxygen vacancies. "V_4_" represents one of the interface oxygen vacancies with black ball, and "V_10_" represents one of the surface oxygen vacancies with black ball.


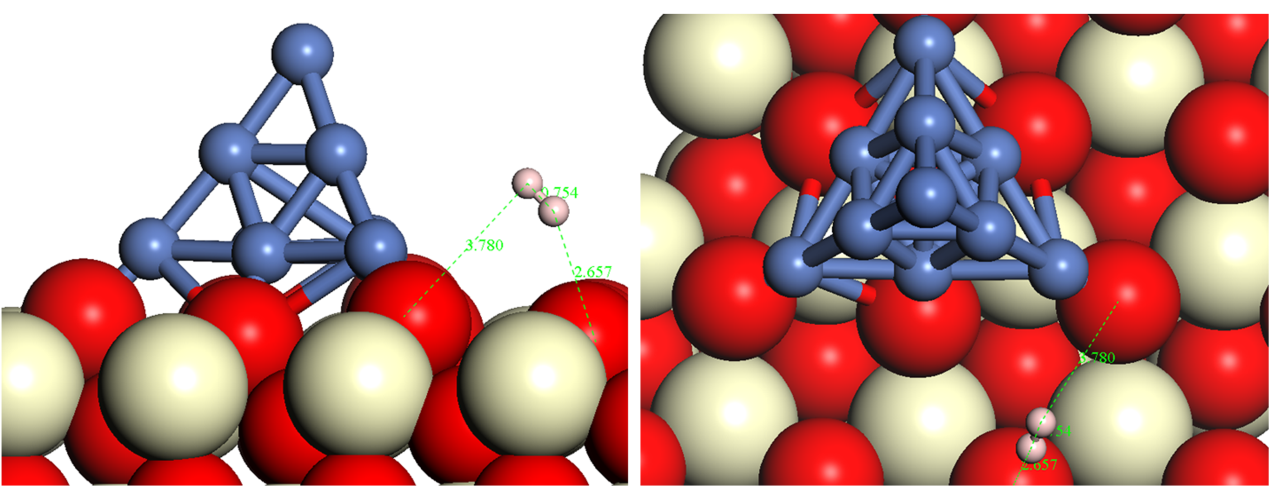


**Figure S3** H_2_ absorbed on the ceria at an angle with the H-H bond length of 0.754 Å. The distances from each H atom to the nearest interface and surface oxygen atoms are 3.780 and 2.657 Å, respectively.


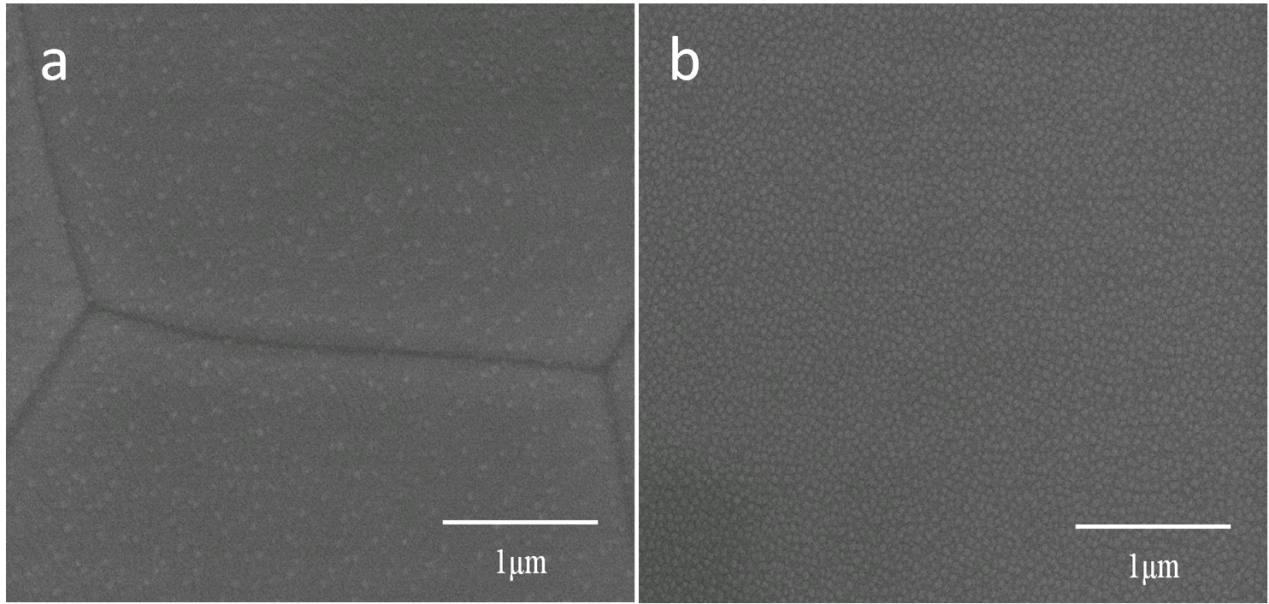


**Figure S4** SEM surface micrographs for the CeO_2_ bars with Ni particles which are prepared by sputtering for (a) 20 s and (b) 80 s.

**Table S1** Ni coverage *θ*, 3PB density *L*_3PB_ (3PB length per unit area), the total reaction rate constant *$k_{chem}$* and the rate constant related to nickel *k*_Ni_ at 800 °C.

|  | CeO_2_ | CeO_2_-Ni20 | CeO_2_-Ni40 | CeO_2_-Ni80 |
| --- | --- | --- | --- | --- |
| *θ* (-) | 0 | 0.0012(3) | 0.0021(2) | 0.0046(6) |
| *L*_3PB_ ($\mu$m^-1^) | 0 | 0.1638±0.0356 | 0.3042±0.0323 | 0.8415±0.1296 |
| *K*_chem_  (10^-5^ cm⋅s^-1^) | 0.72 | 6.03 | 10.2 | 39.1 |
| *k*_Ni_  (10^-5^ cm⋅s^-1^) | 0 | 5.31 | 9.48 | 38.3 |
| *k*_Ni_/*k*_ceria_ | 0 | 7.37 | 12.1 | 53.2 |
| *k*_Ni_/*k*_chem_ | 0 | 0.88 | 0.93 | 0.98 |
